# Uneventful COVID-19 Infection and Vaccination in a Cohort of Patients with Prior Myocarditis

**DOI:** 10.3390/vaccines11121742

**Published:** 2023-11-22

**Authors:** Anna Baritussio, Andrea Silvio Giordani, Cristina Basso, Cristina Vicenzetto, Giulia Lorenzoni, Matteo Gasparin, Sabino Iliceto, Bruno Scarpa, Dario Gregori, Renzo Marcolongo, Alida Linda Patrizia Caforio

**Affiliations:** 1 Cardiology, Department of Cardiac, Thoracic, Vascular Sciences and Public Health, Padua University Hospital, University of Padua, 35128 Padua, Italy; anna.baritussio@aopd.veneto.it (A.B.); andreasilvio.giordani@aopd.veneto.it (A.S.G.); cristina.vicenzetto@aopd.veneto.it (C.V.); sabino.iliceto@unipd.it (S.I.); renzo.marcolongo@aopd.veneto.it (R.M.); 2 Cardiac Pathology, Department of Cardiac, Thoracic, Vascular Sciences and Public Health, University of Padua, 35128 Padua, Italy; cristina.basso@unipd.it; 3 Unit of Biostatistics, Epidemiology and Public Health, Department of Cardiac, Thoracic, Vascular Sciences, and Public Health, University of Padua, 35128 Padua, Italy; giulia.lorenzoni@unipd.it (G.L.); dario.gregori@ubep.unipd.it (D.G.); 4 Department of Statistical Sciences, University of Padua, 35121 Padua, Italy; matteo.gasparin.1@phd.unipd.it (M.G.); bruno.scarpa@unipd.it (B.S.); 5 Department of Mathematics “Tullio Levi Civita”, University of Padua, 35131 Padua, Italy

**Keywords:** myocarditis, myocarditis relapse, COVID-19 infection, COVID-19 vaccination

## Abstract

Myocarditis has in rare cases been associated with COVID-19 infection and has emerged as a possible rare side effect of vaccination with anti-COVID-19 messenger RNA vaccines. However, little is known about possible COVID-19 infection- and/or vaccination-related myocarditis relapse in patients with previous clinically suspected or biopsy-proven myocarditis. Myocarditis may relapse, particularly in females with immune-mediated/autoimmune features and a predisposing immunogenetic background. We aimed to assess the prevalence of myocarditis relapse during the COVID-19 outbreak and following COVID-19 vaccination in a cohort of patients with prior myocarditis. We included in the analysis myocarditis patients on active follow-up, for whom COVID-19 infection and vaccination statuses were known, and collected data on clinical, laboratory and echocardiographic findings, and myocarditis relapse. We enrolled 409 patients, of whom 114 (28%) reported COVID-19 infection and 347 (85%) completed the vaccination scheme. Only one patient, having COVID-19 infection before the vaccination campaign started, was admitted to hospital because of pneumonia; the remaining patients had an uneventful COVID-19 infection course, with only mild symptoms. No myocarditis relapse was recorded following COVID-19 infection or vaccination. Moreover, the frequency of new myocarditis cases following the COVID-19 outbreak was not different compared to the three-year period preceding the COVID-19 era. In conclusion, in our cohort of patients with prior myocarditis, both COVID-19 infection and vaccination were uneventful.

## 1. Introduction

Clinically suspected myocarditis has in rare cases been associated with COVID-19 infection and reported following vaccination with messenger RNA (mRNA) anti-COVID-19 vaccines [1]. The reported cases of myocarditis related to mRNA anti-COVID-19 vaccines initially raised great concern, both among physicians and vaccine recipients (patients and healthy subjects). In fact, at the beginning of the vaccination campaign, an increased number of myocarditis, pericarditis and myopericarditis cases was reported, mainly following the second dose of mRNA anti-COVID-19 vaccines, especially in young males [1,2,3,4]. The absence of histological confirmation of myocarditis [5] in most of the published studies, however, makes it difficult to demonstrate a cause–effect relationship between myocarditis and COVID-19 infection or vaccination. Moreover, the absence of a virological search for known or suspected new cardiotropic agents makes it even harder to prove the causative association in COVID-19-related myocarditis. Myocarditis may relapse, particularly in females with immune-mediated/autoimmune features, heart failure presentation, fulminant onset, biventricular dysfunction and a predisposing immunogenetic background [6,7]. Patients with previous clinically suspected or biopsy-proven myocarditis [5,6,7] might in principle be highly predisposed to reactivation/relapses following COVID-19 infection or anti-COVID-19 mRNA vaccination due to a “second hit” phenomenon. To the best of our knowledge, so far, no data have been published on the risk of developing COVID-19-associated myocarditis or post-mRNA anti-COVID-19 vaccine myocarditis in patients with known history of myocarditis. We report the experience of our center, a regional hub for myocarditis included in the European Reference Network (ERN) GUARD-Heart and comprising 1039 patients. Our aim was to explore the effects of COVID-19 infection and vaccination in our cohort of patients with prior clinically suspected or biopsy-proven myocarditis.

## 2. Materials and Methods

We included in this analysis patients with history of clinically suspected or biopsy-proven myocarditis, actively followed up in our outpatient clinic, and for whom COVID-19 infection and vaccination statuses were known (study protocol number 0021857).

Clinically suspected myocarditis was defined according to the World Health Organization and European Society of Cardiology criteria [5] as follows: ≥1 clinical presentation suggestive of myocarditis and ≥1 diagnostic criterion from different categories (ECG, increased myocytolitic enzymes, morpho-functional abnormalities at cardiac imaging and tissue characterization on CMR) in the absence of angiographically detectable coronary artery disease and known pre-existing cardiovascular disease or extra-cardiac causes that could explain the syndrome; in asymptomatic patients, ≥2 diagnostic tests were needed to diagnose clinically suspected myocarditis. Endomyocardial biopsy (EMB) was performed as clinically indicated according to expert position papers [8,9,10] by obtaining 4–6 myocardial samples, 1–2 mm in size, from the right ventricle [8,9]; one or two frozen EMB specimens per patient were used for polymerase chain reaction (PCR) and reverse transcriptase PCR analysis and for detection of cardiotropic viruses’ genomes simultaneously with histological analysis [10]. EMB-proven myocarditis was defined by histological (Dallas criteria), immunohistochemical (≥14 leucocytes/mm^2^ including up to 4 monocytes/mm^2^ with the presence of CD3-positive T-lymphocytes ≥7 cells/mm^2^) and molecular criteria (search of cardiotropic viruses’ genomes) [5]. A myocarditis relapse was defined following the above-mentioned criteria [5], based upon patients’ symptoms, troponin levels and new ECG or morpho-functional abnormalities shown in cardiac imaging.

For all patients enrolled in this study, we collected data regarding COVID-19 infection and vaccination statuses (start of vaccination campaign in Italy: 1 January 2021). Clinical, electrocardiographic, imaging and laboratory data were collected at myocarditis diagnosis and at each follow-up visit.

### Statistical Analysis

Descriptive variables were reported as mean ± SD; categorical variables were reported as absolute number and percentage. Pearson’s chi-square test with correction for continuity and a *t*-test with Welch’s correction were used for categorical and continuous variables, respectively. A log likelihood ratio test was used to compare the rates of new myocarditis diagnosis during the COVID-19 pandemic and in the preceding three-year period. A pairwise *t*-test was used to compare characteristics of infected and vaccinated patients at the last follow-up before the COVID-19 pandemic and at the last available follow-up. A *p*-value < 0.05 was considered significant. The analyses were performed using R software version 4.3.1 (R Core Team, 2023, A Language and Environment for Statistical Computing. Vienna, Austria: R Foundation for Statistical Computing).

## 3. Results

We included in the analysis 409 patients with prior myocarditis (mean age 47 ± 17, male 69%, biopsy-proven myocarditis 36–91% lymphocytic, mean echocardiographic LVEF at myocarditis diagnosis 50 ± 14% and mean right ventricular fractional area change at myocarditis diagnosis 42 ± 10%). Of these, 114 (28%) patients reported COVID-19 infections and 347 (85%) completed the COVID-19 vaccination scheme. Two-thirds of patients received an mRNA vaccine (mainly Pfizer-BioNTech). Sixty-five percent of patients developed COVID-19 infection following vaccination; eleven percent of patients developed COVID-19 infection more than once. We found similar rates of COVID-19 infection among vaccinated and non-vaccinated patients (27% were infected among the vaccinated patients vs. 32% among the non-vaccinated patients, *p* = 0.50). The median time between myocarditis diagnosis and COVID-19 infection did not differ between vaccinated and non-vaccinated patients (1701 vs. 1271 days, respectively, *p* = 0.29). Only one patient, having a COVID-19 infection before the vaccination campaign started, was admitted to hospital because of pneumonia; all remaining patients had an uneventful COVID-19 infection course, with only mild symptoms.

Clinical and imaging characteristics of patients are reported in Table 1. Patients infected with COVID-19 were younger and less frequently female. We found no difference in cardiovascular risk factors between patients who were infected with COVID-19 and those who were not (systemic arterial hypertension: 9% vs. 15%, *p* = 0.19; diabetes: 2% vs. 2%, *p* = 1.00). Patients who were infected were less likely to have left-sided heart failure at myocarditis diagnosis (12% vs. 21% among patients who did not have COVID-19 infections, *p* = 0.05), while there was no difference between infected and non-infected patients with regard to other cardiac symptoms (chest pain: 42% vs. 40%, *p* = 0.86; syncope: 9% vs. 4%, *p* = 0.12; palpitations: 13% vs. 19%, *p* = 0.33). We found no difference in cardiovascular risk factors between vaccinated and non-vaccinated patients (systemic arterial hypertension: 13% vs. 16%, *p* = 0.7; diabetes: 2% vs. 0, *p* = 0.48); likewise, there was no difference between these two groups with regard to cardiac symptoms at myocarditis diagnosis (chest pain: 40% vs. 41%, *p* = 1.00; syncope: 5% vs. 7%, *p* = 0.76; palpitations: 18% vs. 17%, *p* = 1.00). There was no difference in biventricular function at diagnosis according to both infection and vaccination status; likewise, there was no difference with regard to the frequency of anti-heart autoantibodies (AHA) positivity. There was a trend toward a higher rate of active immune-suppressive treatment among myocarditis patients receiving the vaccination (23% vs. 11%, *p* = 0.06).

We did not record any case of myocarditis relapse during or following COVID-19 infection. Likewise, no case of myocarditis relapse was reported in our study cohort following vaccination, early after vaccination (within three weeks from first or second dose) or later. Moreover, we found no difference in the rate of new myocarditis cases following the COVID-19 outbreak in Italy (1 March 2020 to 31 May 2023, *n* = 115) as compared to the three-year period preceding the beginning of the pandemic (1 January 2017 to 29 February 2020, *n* = 114) (*p* = 0.30). With regard to the new myocarditis cases, out of 54 patients with biopsy-proven myocarditis in the three years preceding the COVID-19 outbreak in Italy, 51 had lymphocytic, 2 had eosinophilic (1 within the conundrum of eosinophilic granulomatosis with polyangiitis and 1 within the conundrum of polymyositis) and 1 had giant-cell myocarditis; 8 patients had viral myocarditis (all lymphocytic), 4 due to Adenovirus, 2 due to Human Herpes-virus 6, 1 due to Cytomegalovirus and 1 to Parvovirus B19 (PVB19). Among the new myocarditis cases following the COVID-19 outbreak in Italy, 26/30 had lymphocytic, 3 had eosinophilic (1 within the conundrum of eosinophilic granulomatosis with polyangiitis and 2 viral) and 1 had polymorphic myocarditis; 5 patients had viral myocarditis (3 lymphocytic and 2 eosinophilic cases), 4 due to PVB19 and 1 due to Epstein–Barr virus. There was no difference in the histological type of new myocarditis cases following the COVID-19 outbreak in Italy and in the three years preceding it (*p* = 0.22); there was also no difference in the rate of viral myocarditis between the two time intervals considered (5/30 vs. 8/54, *p* = 0.82). Of the new myocarditis cases diagnosed during the COVID-19 pandemic, none were associated with COVID-19 infection: all patients were tested for COVID-19 through nasal swabs and SARS-CoV-2 virus was searched for on myocardial tissue using molecular virological techniques in patients undergoing EMB. Only four patients presented with myocarditis temporally associated with the first dose of mRNA anti-COVID-19 vaccination. However, one patient who had respiratory symptoms days before receiving the vaccine tested positive for recent adenoviral infection the week following vaccination (see Table 2), therefore raising the suspicion that myocarditis may be more likely related to adenoviral infection; one patient had biopsy-proven autoimmune virus-negative massive lymphocytic myocarditis with no signs of hypersensitivity; one patient had PVB19 biopsy-proven fulminant lymphocytic myocarditis; and one patient had clinically suspected myocarditis with associated pericarditis three weeks following the first mRNA vaccine dose, with a benign clinical course (mild increase in troponin levels and preserved biventricular function). Clinical, laboratory and imaging data of these four patients are summarized in Table 2. All patients showed normal biventricular function at the last follow-up with normalization of troponin I levels.

Finally, no clinically significant differences were found when comparing patients’ characteristics between the follow-up prior to the COVID-19 outbreak and the last follow-up, irrespective of infection and vaccination status (Table 3).

## 4. Discussion

Clinically suspected myocarditis has in rare cases been associated with COVID-19 infection, although a direct causative role for SARS-CoV-2 is still controversial [1]. In fact, alternative immune-mediated mechanisms, in particular virus-driven cytokine storm or naturally occurring viral or autoimmune myocarditis, cannot be excluded [1]. The potential mechanisms leading to COVID-19-related myocarditis are still debated. First, although direct infection was presumed to be the cause of COVID-19-related viral myocarditis, no autopsy studies have found histologically proven myocarditis associated with SARS-CoV-2 via molecular methods [11], with viral genome/particles being found in endothelial cells and macrophages, but not in cardiomyocyte; moreover, only low levels of virus were detected in animal models’ hearts. Furthermore, administration of pro-inflammatory cytokines alone was not sufficient to induce myocarditis in animal models [11]. Myocarditis has also been reported as a possible rare side effect after vaccination with messenger RNA (mRNA) COVID-19 vaccines, especially following the second dose [1,2,3,4,12], and generally presents in mild form and with short duration [11]. Many causes of vaccine-related myocarditis have been hypothesized, including cytokine storm, hypersensitivity and molecular mimicry between cardiac autoantigens, including myosin heavy chain and the virus spike protein [12,13,14]. Overall, however, the major limitations to proving a causative link between COVID-19 infection or vaccination and myocarditis relate to the lack of endomyocardial biopsy. In fact, endomyocardial biopsy is the only test which provides a diagnosis of certainty for myocarditis, using established histological and immunohistological criteria, as well as an etiological diagnosis and molecular virological techniques applied to myocardial tissue [1,5], as per the European Society of Cardiology criteria [5]. Therefore, the results of published studies on COVID-19 infection- or vaccination-related myocarditis should be cautiously interpreted. First, in the absence of endomyocardial biopsy, it is not possible to be sure that there is histological myocarditis [5]. Second, it is not possible to identify both the causative agent and the mechanism of myocarditis in the absence of a virological search for known cardiotropic viruses (such as Enterovirus, Adenovirus) and for suspected new agents, i.e., SARS-CoV-2, by polymerase chain reaction, in situ hybridization and/or electron microscopy on endomyocardial biopsy [5]. Conversely, published studies on suspected myocarditis temporally associated with COVID-19 infection or vaccination use the Brighton Collaboration Myocarditis Case Definition (CDC), in which endomyocardial biopsy with virological assessment is not mandatory and even the reference non-invasive cardiac imaging test, cardiovascular magnetic resonance (CMR) with tissue characterization sequences, is optional [15,16,17,18]. Data from 23 centers and over 56,000 COVID-19 admissions show an estimated prevalence of acute myocarditis, defined according to the less stringent Brighton Collaboration Myocarditis Case Definition, of 0.2–0.4% [17]. However, there are no reliable estimates of the rate of clinically suspected myocarditis before the COVID-19 pandemic to compare with the scarce cases of COVID-19-related clinically suspected myocarditis. Therefore, it remains unproven whether the rare reports of suspected myocarditis associated with COVID-19 represent true myocarditis and are indeed caused by the SARS-CoV-2 virus, by other viruses, or by an immune-mediated non-infectious mechanism. As of June 2021, the reported cases of myocarditis (that included, however, myocarditis, pericarditis and myopericarditis cases) following anti-COVID-19 vaccination in the United States of America, based on the Brighton Collaboration Myocarditis Case Definition, were higher in younger individuals (highest in the age range 12–17 years, with 56–69 cases per million vaccine doses as opposed to 3–4 cases per million vaccine doses in individuals aged ≥ 30 years) of male gender [2,3,4]. The rarely reported cases of suspected myocarditis with a temporal relation to COVID-19 vaccines may be due to naturally occurring myocarditis, viral or immune-mediated, which is indeed more frequent in young males. In keeping with this, in a recent German study on suspected COVID-19 vaccine-associated myocarditis patients, endomyocardial biopsy revealed that a sizable number of patients did not have histologically confirmed myocarditis, others had viral myocarditis unrelated to SARS-CoV-2 and a majority had immune-mediated myocarditis [19]. In keeping with these findings, we found that the only two new cases of histologically proven myocarditis diagnosed following the first dose of COVID-19 mRNA vaccine failed to show vaccine-induced hypersensitivity features. It is also interesting to note that out of four patients referred with a first episode of myocarditis following anti-COVID-19 vaccination, only one was male and none were in the age range more frequently affected by myocarditis temporally related to vaccine administration. Myocarditis not related to COVID-19 infection or vaccination is most frequent in young males with a pseudo-infarct presentation, increased troponins, preserved biventricular function and often increased systemic inflammation indexes, such as reactive C protein [1]. However, this is also the most frequently observed naturally occurring myocarditis presentation and it usually has a benign course with spontaneous resolution. This is similar to the clinical course of the rare COVID-19 vaccine-associated cases [20]. Therefore, it is possible that what has been reported as temporally related to the vaccine simply represents naturally occurring viral or immune-mediated benign myocarditis in young males. In keeping with this interpretation, our data clearly show equivalent frequencies of new myocarditis diagnosis before the COVID-19 era, and during the pandemic and vaccination period, without an excess of new cases. Similar data have been reported in a smaller series of patients with prior myocarditis [21]. Moreover, we found no difference in the histological type of myocarditis during the COVID-19 pandemic and in the three years preceding its outbreak, nor any difference in the frequency of viral genome detection of endomyocardial biopsies.

Despite having an uneventful course in half of cases, myocarditis may indeed relapse, particularly in females with immune-mediated/autoimmune features, heart failure presentation, fulminant onset, biventricular dysfunction and a predisposing immunogenetic background [6]. As the most accredited explanation of COVID-19-related myocarditis relies on the pro-inflammatory response to myocardial damage activating an autoimmune response in susceptible subjects, patients with prior myocarditis might be at increased risk of myocarditis relapse following COVID-19 infection or anti-COVID-19 (particularly mRNA) vaccination due to a “second hit” phenomenon. To the best of our knowledge no data have been published so far investigating the risk of developing COVID-19-associated or post-mRNA vaccine myocarditis in patients with known prior myocarditis.

The main result of the study presented here is the uneventful course of COVID-19 infection and vaccination in a cohort of patients with prior clinically suspected or biopsy-proven myocarditis. Only one unvaccinated patient presented a complicated COVID-19 infection requiring hospitalization for pneumonia, but this did not lead to a myocarditis relapse or to a worsening of cardiac function. Our hospital was among the first to face the COVID-19 pandemic in Europe and our center is a regional hub for myocarditis, included in the European Reference Network (ERN) GUARD-Heart, comprising 1039 patients with myocarditis. We therefore believe that our cohort could serve as a real-world representation of the risk of myocarditis relapse following COVID-19 infection or vaccination in patients with a prior myocarditis. The rate of COVID-19 vaccination in our studied cohort (85%) was in line with Italian national estimates (90%; https://www.governo.it/it/cscovid19/report-vaccini/, accessed on 11 October 2023). Conversely, the rate of COVID-19 infection in our cohort, which was more prevalent among males in keeping with data in the literature [11], was lower than that reported in Italy. This could be due to a selection bias, as we have only included in the present analysis patients on active follow-up, both before and during the pandemic, for whom COVID-19 infection status was recorded. Moreover, this could also be due to the fact that, having had prior myocarditis, patients were advised to pay particular attention to preventive strategies, especially in the pre-vaccination era. None of the patients from our studied cohort developed a myocarditis relapse following COVID-19 infection or vaccination, either early or later, and we also found no difference in the prevalence of new myocarditis cases during the COVID-19 pandemic as compared to the preceding three-year period (1 March 2020 to 31 May 2023 vs. 1 January 2017 to 29 February 2020). We recorded no cases of myocarditis related to or associated with COVID-19 infection and only one case of myocarditis was temporally associated with the first dose of mRNA vaccine. Finally, we found no clinically relevant difference in clinical and imaging characteristics following COVID-19 infection or vaccination. Our results, therefore, do not seem to support a clinically relevant role of COVID-19 infection or mRNA vaccination in triggering myocarditis relapse/reactivation in patients with prior known myocarditis. We therefore encouraged our patients, especially the biopsy-proven cohort receiving immune-suppressive treatment, to receive COVID-19 vaccination, in keeping with a recent consensus document by the Heart Failure Association of the ESC and the ESC Working Group on Myocardial and Pericardial Diseases [20]. Our findings are in keeping with a recent retrospective population-based cohort study from linkable administrative health databases in the Lombardy region, showing no increased incidence of myocarditis either during or after the SARS-CoV-2 pandemic peak, or indeed during or after the vaccine campaign, with a non-significant trend for a reduced incidence from 2019 to 2021, suggesting that social distancing and facial masking might have indeed even reduced the occurrence of infectious myocarditis. In addition, again in keeping with our results, these authors did not observe a higher frequency of myocarditis temporally associated with the high-risk period post-vaccination in patients with previous history of myocarditis or with autoimmune diseases [22].

Finally, we must emphasize that it cannot be excluded that the clinical and diagnostic findings of COVID-19-related myocardial damage, especially by means of Troponin increase, represent ischemic myocardial damage [23] rather than myocarditis. Myocardial necrosis is not equivalent to biopsy-proven myocarditis; chest pain, dyspnea, troponin increase and electrocardiographic and imaging abnormalities may be due to ischemia, endothelial dysfunction, thrombotic events and hypoxia, all of which are very common in COVID-19 infection [1,11,24]. The COVID-Heart study recently compared the CMR findings of 342 COVID-19-positive patients with increased Troponin levels to two control groups, 113 COVID-19-negative patients with negative Troponin levels but similar comorbidities and 64 hospitalized COVID-19-positive patients with negative Troponin levels [24]. Despite showing twice as many cardiac abnormalities on CMR as compared to either control group, the excess late gadolinium enhancement (LGE) found in the COVID-19-positive Troponin-positive group was attributed to myocardial infarction and micro-infarcts, while there was no significant difference among the three groups with regard to non-ischemic LGE pattern.

## 5. Conclusions

In conclusion, we found that in patients with previous clinically suspected or biopsy-proven myocarditis both COVID-19 infection and anti-COVID-19 mRNA vaccination were uneventful. Ours and other recent findings suggest a critical reappraisal of the clinical significance of the reported associations between COVID-19 infection or mRNA vaccination and myocarditis onset; the rare, reported cases of suspected myocarditis associated with COVID-19 infection or anti-COVID-19 vaccines might represent temporal association rather than causation, or non-specific cytokine-mediated myocardial injury [13,14,15].

## Figures and Tables

**Table 1:** Patients’ characteristics according to COVID-19 infection status and vaccination status.

	COVID-19 Infection		*p*-Value	COVID-19 Vaccination		*p*-Value
	Yes (*n* = 114)	No (*n* = 295)		Yes (*n* = 347)	No (*n* = 62)	
Age, years	41 ± 16	47 ± 17	0.002	45 ± 17	43 ± 16	0.30
Gender, female	24 (21)	104 (35)	0.006	106 (31)	20 (33)	0.85
Extra-cardiac immune-mediated diseases	21 (18)	63 (21)	0.60	70 (20)	14 (23)	0.79
Allergy	24 (21)	79 (27)	0.30	92 (27)	11 (18)	0.22
Clinical presentation			0.04			0.78
Pseudo-infarct	79 (32)	169 (68)		211 (85)	37 (15)	
Heart failure	20 (19)	86 (81)		91 (86)	15 (14)	
Arrhythmia	15 (27)	40 (72)		45 (82)	10 (18)	
NYHA class at diagnosis			0.03			0.46
I	99 (87)	226 (13)		273 (79)	52 (21)	
II-IV	15 (76)	69 (23)		74 (84)	10 (16)	
BBB at diagnosis			0.36			0.70
right	8 (33)	16 (67)		20 (83)	4 (17)	
left	4 (17)	19 (83)		21 (91)	2 (9)	
LVEF Echo, %	52 ± 18	52 ± 17	0.95	52 ± 17	53 ± 17	0.59
FAC, %	43 ± 9	41 ± 9	0.20	42 ± 9	44 ± 9	0.13
Biopsy-proven myocarditis	26 (17)	123 (83)	0.33	113 (87)	17 (13)	0.67
Positive viral PCR on EMB	8 (33)	16 (66)	0.29	20 (83)	4 (17)	>0.99
Positive AHA	46 (29)	114 (71)	0.71	141 (86)	23 (14)	0.21
Immunosuppressive therapy	18 (17)	67 (23)	0.21	78 (23)	7 (11)	0.06

Data are expressed as mean ± SD and *n* (%). AHA: anti-heart autoantibodies; BBB: bundle branch block; EMB: endomyocardial biopsy; FAC: fractional area change; LVEF: left ventricular ejection fraction; NYHA: New York Heart Association; PCR: polymerase chain reaction.

**Table 2:** Clinical characteristics of the four new myocarditis cases temporally associated with COVID-19 vaccination.

	Case 1	Case 2	Case 3	Case 4
Age at diagnosis, years	21	43	55	20
Gender	Male	Female	Female	Female
Days from mRNA vaccination to symptoms onset	6 *	6	14	21
COVID-19 nasal swab	Negative	Negative	Negative	Negative
Clinical Presentation	Infarct-like	HF	HF	Infarct-like
Peak Troponin I level, ng/L	4068	331	13,846	Raised Troponin T
AHA status	OS, mildly positive	OS, mildly positive	Negative	not performed
LVEF Echo at diagnosis, %	56	45	10 **	55
FAC at diagnosis, %	42	mild reduction	20	37
Biopsy-proven	No	Yes	Yes	No
Histological type	-	Lymphocytic (no eosinophilic, giant cell, and histiocytic features)	Lymphocytic, PVB19 > 500 copies/ug on EMB (no eosinophilic, giant cell, and histiocytic features)	-
PCR for SARS-CoV-2 genome on EMB	-	Negative	Negative	-
Immune-suppressive treatment for myocarditis	-	Yes	No	-
LVEF on CMR at diagnosis, %	54	mild reduction	64 ***	normal
RVEF on CMR at diagnosis, %	61	mild reduction	67	normal
Presence of myocardial edema	Mid-wall pattern, basal inferior and inferolateral walls	Diffuse	Diffuse	Increased T2mapping of the antero-lateral wall
Presence of myocardial LGE	Mid-wall pattern, basal inferior and inferolateral walls	Diffuse subendocardial	Mid-wall/epicardial pattern, basal-mid inferior, basal-apical lateral, mid-apical septal walls	Mid-wall pattern, basal-mid anterior and anterolateral wall
Last follow-up, years from diagnosis	2	1.5	1.5	2
Troponin I level at last follow-up	Normal	Normal	Normal	Normal
LVEF Echo at last follow-up, %	58	63	64	55
FAC at last follow-up, %	43	43	46	47

CRP: C-reactive protein; EMB: endomyocardial biopsy; HF: heart failure; LGE: late gadolinium enhancement; OS: organ-specific; PVB19: Parvovirus B19; for the remaining abbreviations see Table 1. * The patient developed a cough 5 days before vaccination; on the day of vaccination, he became febrile with a sore throat and limb erythematous nodules; IgG anti-Adenovirus tested positive 8 days following vaccination (roughly 2 weeks since the beginning of respiratory symptoms) with no evidence of Adenovirus DNA on patients’ serum; these tests were deemed to be consistent with recent adenoviral infection by our Infectious Disease Specialist. ** Refractory cardiogenic shock requiring extra-corporeal membrane oxygenator (ECMO). *** CMR was performed after ECMO removal, 3 weeks after presentation.

**Table 3:** Comparison of COVID-19-infected and vaccinated patients’ characteristics at last follow-up in 2019 (before COVID-19 outbreak) as compared to the last follow-up available.

	COVID-19 Infected Patients (*n* = 114)		*p*-Value	COVID-19 Vaccinated Patients (*n* = 347)		*p*-Value
	Last Follow-Up before COVID-19 Outbreak	Last Available Follow-Up		Last Follow-Up before COVID-19 Outbreak	Last Available Follow-Up	
NYHA class			0.68			0.56
I	65	67		217	221	
II-IV	4	2		16	12	
BBB	6	5	>0.99	29	31	0.89
LVEF Echo, %	63 ± 8	61 ± 8	0.01	62 ± 9	59 ± 10	<0.01
RVEDA, cm^2^	18.4 ± 4.7	19.4 ± 4.9	0.02	18.6 ± 4.7	19.4 ± 4.9	0.004
FAC, %	50.3 ± 7.6	46.2 ± 7.9	0.003	50.9 ± 8.2	45.4 ± 7.8	<0.01

RVEDA: right ventricular end-diastolic area. Or the other abbreviations see Table 1.

## Data Availability

Data are available upon reasonable request to the corresponding author.
